# Supplementary material for: Identification of Freezing-Responsive microRNAs and Their Targets in Chinese Jujube by Small RNA and Degradome Sequencing
Source: Int J Mol Sci. 2026 Sep 4;27(17):7912. doi: 10.3390/ijms27177912 (PMC13566890; doi:10.3390/ijms27177912)
Supplement: Supplementary file 1 [file ijms-27-07912-s001.zip › Supplementary Material S2/novel_miR_153.pdf]

Provisional ID	:	novel_miR_153
Score total	:	252
Score for star read(s)	:	3.9
Score for read counts	:	246.4
Score for mfe	:	0.1
Score for randfold	:	1.6
Score for cons. seed	:	
Total read count	:	495
Mature read count	:	460
Loop read count	:	34
Star read count	:	1

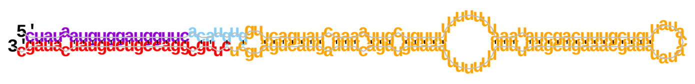

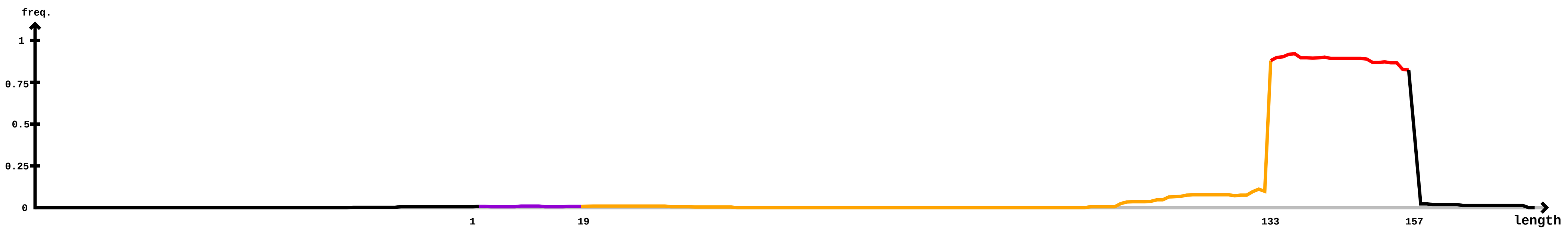

Star   Mature

```
5'- aaguugaagcaauauagacauaaauaaaaacuaaaagauauaaauaaaaacuaaaagauaugugggauaaaacuauaaauguggaugguucacaugugguucaguaucaaaauugcuguuuuuuuuuuaaauaucgacuuugcuguuauacauauauagcaaagucgauuuuuuuuuuuuuaaauguugacuuuaguacugaugcucuugcggaccgucuguaucauagcuucguucaagauaugugagaua -3' obs
    aaguugaagcaauauagacauaaauaaaaacuaaaagauauaaauaaaaacuaaaagauaugugggauaaaacuauaaauguggaugguucacaugugguucaguaucaaaauugcuguuuuuuuuuuaaauaucgacuuugcuguuauacauauauagcaaagucgauuuuuuuuuuuuuaaauguugacuuuaguacugaugcucuugcggaccgucuguaucauagcuucguucaagauaugugagaua     exp
```

alignment	reads	mm	sample
(.((((.(.((((.((.((((.(((((((((((((.((.(.(..(((((((.(((.(((.(((((......(((.((((((((((((((........))))))))))))).))).......))))).))).))).)))))))..).).)).))))))))))))).)))).)).)))).).)))).)......			
uaaaagauaugugggauaaaacu	1	0	JCK-3
augugggauaaaacuauaaaugugg	1	0	JCK-3
augugggauaaaacuauaaaugugg	1	0	JCK-2
cuauaaauguggaCgguuc	1	1	LT-2
guggaugguucacaugugguucagu	1	0	LT-2
guNgaugguucacaugugguucagu	1	1	LT-2
uucacaugugguucaguauca	1	0	JCK-1
caugugguucaguaucaaaauugc	1	0	JCK-1
caugugguucaguaucaaaauugc	1	0	JCK-2
uuuuuaaauguugacuuuaguacu	1	0	JCK-2
uuuuuaaauguugacuuuaguacu	2	0	JCK-3
aaauguugacuuuaguacugaugc	4	0	JCK-2
aaauguugacuuuaguacugaugc	4	0	JCK-3
aaauguugacuuuaguacugaugc	2	0	JCK-1
aauguugacuuuaguacugaugcu	3	0	LT-1
aauguugacuuuaguacugaugcu	2	0	JCK-2
auguugacuuuaguacugaugcu	1	0	LT-3
uugacuuuaguacugaugcA	1	1	JCK-2
ugacuuuaguacugaugcucuugc	1	0	JCK-3
ugacuuuaguacugaugcucuugc	4	0	JCK-2
acuuuaguacugaugcucuug	1	0	JCK-2
acuuuaguacugaugcucuugc	3	0	JCK-2
acuuuaguacugaugcucuugc	3	0	JCK-1
acuuuaguacugaugcucuugc	2	0	JCK-3
cuuuaguacugaugcucuugcgg	1	0	LT-3
uuuaguacugaugcucuugcggac	1	0	LT-3
uuaguacugaugcucuugcggacc	2	0	JCK-3
uuaguacugaugcucuugcggacc	1	0	JCK-1
uuaguacugaugcucuugcggacc	1	0	JCK-2
uaguacugaugcucuugcgga	1	0	JCK-2
augcucuugcggaccgucugu	2	0	JCK-2
Ccucuugcggaccgucugua	11	1	LCK-3
cucuugcggaccgucuguauc	1	0	JCK-2

Star — Mature

aaguugaagcaauauagacauaaauaaaaacuaaaagauauaaauaaaaacuaaaagauauguggauaaaacuauaauguggaugguucacaugugguucaguaucaaaauugcuguuuuuuuuuuaaauaucgacuuugcuguuauacauauauagcaaagucgauuuuuuuuuuuaaauguugacuuuaguacugaugcucuugcggaccgucuguaucauagcuucguucaagauaugugagaua

cucuugcggaccgucuguaucU	1	1	LT-3
cucuugcggaccgucuguaucaua	2	0	JCK-1
cucuugcggaccgucuguaucaua	3	0	JCK-2
cucuugcggaccgucuguaucaua	1	0	JCK-3
ucuugcggaccgucuguauca	1	0	LT-3
ucuugcggaccgucuguaucauC	1	1	JCK-2
ucuugcggaccgucuguaucauaU	1	1	LT-1
cuugcggaccgucuguauc	1	0	LT-1
Uuugcggaccgucuguauc	3	1	LCK-3
cuugcggaccgucuguauca	1	0	JCK-2
cuugcggaccgucuguaucaua	5	0	JT-3
cuugcggaccgucuguaucaua	5	0	JT-2
cuugcggaccgucuguaucauCgc	1	1	JCK-3
cuugcggacAgucuguaucauagc	1	1	JCK-1
cuugcggaccgucuguaucauagc	86	0	JCK-1
cuugcggaccgucuAuaucauagc	1	1	JCK-3
cuugcgAaccgucuguaucauagc	2	1	JCK-3
cuugcggaccgucuguaucauagc	15	0	LT-3
Uuugcggaccgucuguaucauagc	5	1	LT-2
Uuugcggaccgucuguaucauagc	1	1	LT-3
Uuugcggaccgucuguaucauagc	3	1	LT-1
cuugcggaccgucuguaucauagc	105	0	JCK-3
cNugcggaccgucuguaucauagc	1	1	JCK-2
cuugcggaccgucuguaucauagc	9	0	LT-1
cuugcggCccgucuguaucauagc	2	1	JCK-3
cuugcggaccgucuguaucauagc	10	0	LT-2
cuugcggaccgucuguaucauagc	3	0	JT-1
Uuugcggaccgucuguaucauagc	1	1	JCK-2
Uuugcggaccgucuguaucauagc	1	1	JCK-3
cuugcggaccgucuguaucauagc	3	0	LCK-3
cuugcggaccgucuguaucauagc	159	0	JCK-2
uugcggaccgucuguaucauU	2	1	JCK-3
uugcggaccgucuguaucauU	2	1	JCK-2
uugcggaccgucuguaucauagc	1	0	LT-1
uugcggaccgucuguaucauagc	1	0	JCK-2
uugcggaccgucuguaucauagc	1	0	JCK-3
uugcggaccgucuguaucauagc	2	0	JCK-1
uugcggaccgucuguaucauagcC	1	1	LT-3
ugcggaccgucuguaucauag	1	0	JCK-3
ugcggaccgucuguauAauagc	1	1	LT-3
gcggaccgucuguaucauagc	5	0	LT-2
gcggaccgucuguaucauagc	1	0	LT-1
gcggaccgucuguaucauagc	2	0	LT-3
cggaccgucuguaucauagc	1	0	JCK-2
cggaccgucuguaucauagA	1	1	LT-3
cggaccgucuguaucauagcC	1	1	LT-3
ccgucuguaucauagcuuc	2	0	LCK-3
cgucuguaucauaAcuucguuca	2	1	LT-1
cgucuguaucauagcuucguuca	1	0	LT-1
auagcuucguucaagauaugugag	2	0	JCK-3
auagcuucguucaagauaugugag	3	0	JCK-1
auagcuucguucaagauaugugag	2	0	JCK-2
